# Testosterone replacement therapy and cardiovascular safety in older men: lessons from TRAVERSE and beyond

**DOI:** 10.1007/s40618-026-02945-w

**Published:** 2026-06-27

**Authors:** Daniele Tienforti, Giovanni Terrana, Riccardo Di Geronimo, Elisabetta Perfetto, Luca Spagnolo, Marco Giorgio Baroni, Arcangelo Barbonetti

**Affiliations:** Andrology Unit, Department of Clinical Medicine, Life, Health and Environmental Sciences, University of L’Aquila, Via Vetoio, L’Aquila, 67100 Italy

**Keywords:** Testosterone replacement therapy, Hypogonadism, Cardiovascular safety, TRAVERSE, Atrial fibrillation, Venous thromboembolism, Functional hypogonadism, Erythrocytosis

## Abstract

**Background:**

The cardiovascular safety of testosterone replacement therapy (TRT) in older men with hypogonadism has been debated for over a decade, largely on the basis of underpowered trials and conflicting observational data. The TRAVERSE trial, published in 2023, provided the first adequately powered, placebo-controlled evidence on this question.

**Objectives:**

This review synthesises current evidence on the cardiovascular safety of TRT in older men, with particular attention to the interpretation of TRAVERSE findings, the clinical significance of non-MACE safety signals, and the practical management of organic versus functional hypogonadism.

**Methods:**

A systematic search of PubMed/MEDLINE, Embase, and the Cochrane Central Register of Controlled Trials (CENTRAL) was conducted through March 2026. Approximately 450 articles were identified; 64 met the inclusion criteria and were selected for review. Evidence was appraised according to study design, with RCTs and meta-analyses weighted above observational data.

**Results:**

TRAVERSE demonstrated non-inferiority of TRT versus placebo for major adverse cardiovascular events (MACE) in men with confirmed hypogonadism and elevated cardiovascular risk (HR 0.96, 95% CI 0.78–1.17) [5]. Non-MACE signals — including atrial fibrillation (3.1% vs. 2.4%), pulmonary embolism (2.0% vs. 1.5%), and acute kidney injury (2.3% vs. 1.5%) — were numerically higher in the TRT arm but did not reach statistical significance within the trial. In contrast, large observational cohorts consistently report statistically significant associations between TRT and AF and VTE. Erythrocytosis was the most reproducible adverse effect (17.0% vs. 3.3%, *p* < 0.001). Functional hypogonadism secondary to obesity or metabolic syndrome responds to lifestyle intervention and GLP-1/GIP agonists, with testosterone normalisation in 81.4% at 6 months and 89.5% at 24 months after bariatric surgery [45].

**Conclusions:**

TRT does not increase MACE risk in men with confirmed organic hypogonadism when titrated to physiological levels. Non-MACE signals warrant vigilance rather than contraindication. Metabolic optimisation should precede TRT in functional hypogonadism. Individualised monitoring and careful patient selection remain essential.

## Introduction

Few topics in endocrinology have generated as much uncertainty, and at times as much regulatory instability, as the cardiovascular safety of testosterone replacement therapy (TRT). Over the past fifteen years, clinicians have had to navigate a fragmented evidence base: small, underpowered trials, conflicting observational studies, and regulatory warnings that were themselves based on limited data. The result was a clinical environment in which many men with genuine hypogonadism were denied or delayed treatment on the basis of concerns that the available evidence did not fully support.

A turning point came in 2010, when the TOM trial was stopped early due to an excess of cardiovascular events in the testosterone arm, a finding later attributed, at least in part, to supraphysiological testosterone levels and an unusually high-risk study population [1]. The FDA responded swiftly: in 2015, a cardiovascular warning was mandated across all testosterone products. In the years that followed, clinical practice became more cautious, and many men with clear hypogonadism experienced delays in treatment initiation, often driven by concerns rooted in a relatively limited body of evidence.

The observational studies that followed did little to resolve the uncertainty. Vigen and colleagues reported an increased risk of mortality and cardiovascular events with TRT [2], while Finkle et al. described a higher rate of non-fatal myocardial infarction in the 90 days after testosterone prescription [3]. Both studies attracted substantial methodological criticism, yet their findings were widely cited and contributed to the regulatory climate of caution. Other observational analyses yielded contradictory results, and the field was left without a reliable answer to a clinically urgent question [4].

The TRAVERSE trial provided the first adequately powered attempt to address this question. Published in the New England Journal of Medicine in 2023, it enrolled 5,246 men with confirmed hypogonadism and elevated cardiovascular risk, and demonstrated non-inferiority of TRT versus placebo for major adverse cardiovascular events [5]. The trial also generated important data on non-MACE safety signals (atrial fibrillation, venous thromboembolism, acute kidney injury, and erythrocytosis) that had not been systematically evaluated in prior studies.

This review positions the TRAVERSE findings within a broader clinical framework. Three questions that remain insufficiently resolved are addressed: how to interpret the non-MACE safety signals from TRAVERSE in the context of observational data; how to distinguish between organic and functional hypogonadism and what that distinction means for treatment decisions; and how to apply the available evidence to individual patients with varying risk profiles.

## Search strategy and study selection

This structured narrative review was informed by a systematic search of PubMed/MEDLINE, Embase, and the Cochrane Central Register of Controlled Trials (CENTRAL), last updated in March 2026.

The search strategy combined terms related to testosterone replacement therapy and hypogonadism with those addressing cardiovascular safety. Specifically, the following keyword clusters were used: (“testosterone” OR “androgen” OR “testosterone replacement therapy”) AND (“cardiovascular” OR “MACE” OR “myocardial infarction” OR “stroke” OR “atrial fibrillation” OR “venous thromboembolism” OR “pulmonary embolism” OR “acute kidney injury”) AND (“hypogonadism” OR “testosterone deficiency” OR “functional hypogonadism” OR “late-onset hypogonadism”). Additional searches addressed specific topics including erythrocytosis, bone safety, and alternative treatments for functional hypogonadism.

Studies were considered eligible if they met the following criteria: inclusion of adult men (≥ 18 years) with confirmed or suspected hypogonadism; reporting of cardiovascular or metabolic outcomes in the context of testosterone therapy; publication in English in peer-reviewed journals; and availability of the full text. Both interventional and observational study designs were included, as were systematic reviews, meta-analyses, and clinical practice guidelines.

Studies conducted in women or paediatric populations were excluded, as were case reports, letters, and editorials. Studies focusing exclusively on non-cardiovascular outcomes without reporting safety data were also excluded.

**Fig. 1:**
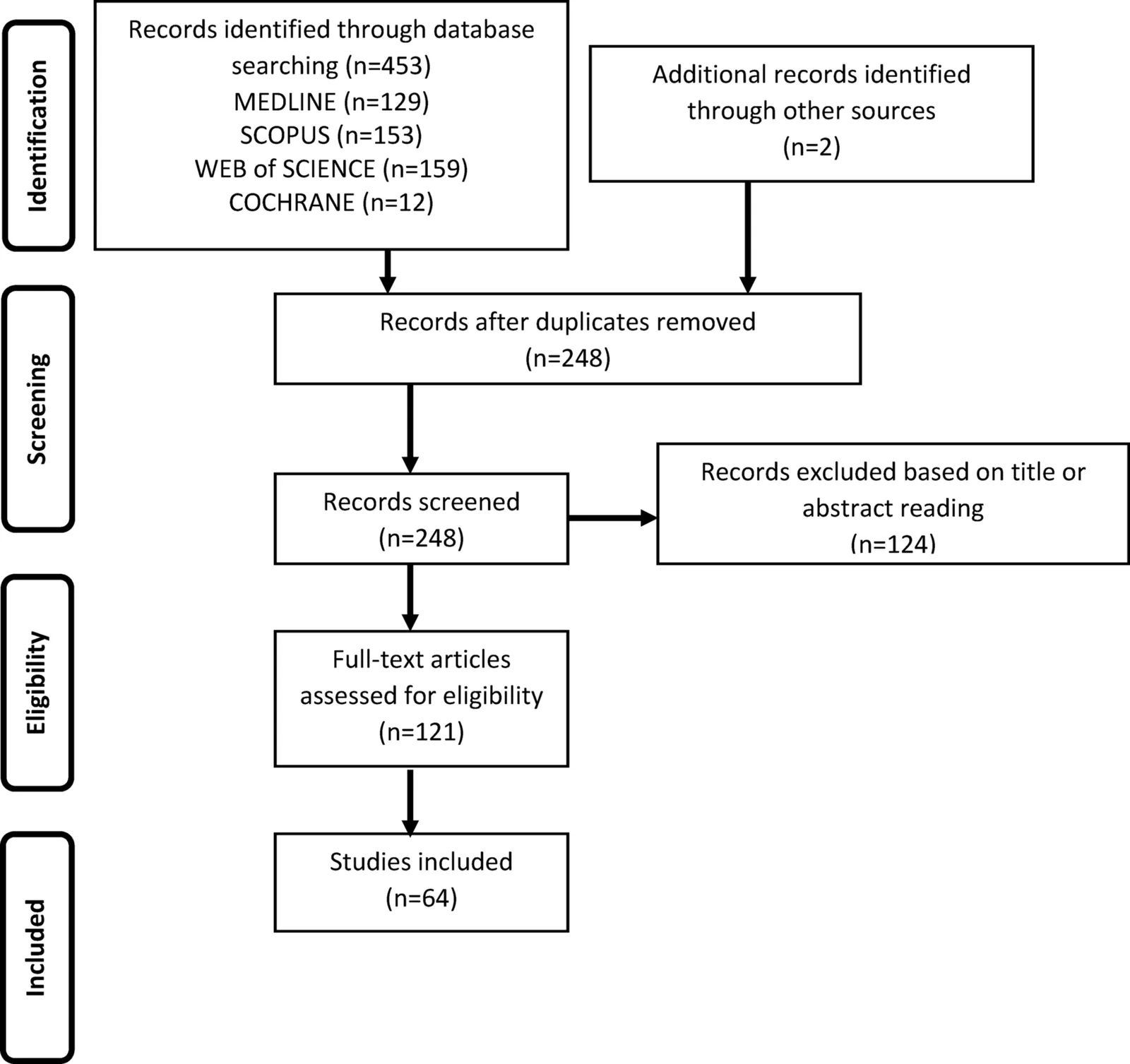
PRISMA flow diagram of study selection

The initial search identified approximately 450 records. After screening titles and abstracts, followed by full-text evaluation against the predefined criteria, 64 articles were included in the final review. The full selection process is summarised in Fig. 1.

In appraising the available evidence, priority was given to randomised controlled trials and their meta-analyses. Within this framework, the TRAVERSE trial represents the highest level of evidence currently available on the cardiovascular safety of TRT. Observational studies were used to contextualise and extend the trial findings, particularly for outcomes that were not systematically assessed in TRAVERSE.

## The TRAVERSE trial: what it established and what it did not

### Design and population

TRAVERSE was a double-blind, placebo-controlled, event-driven non-inferiority trial conducted across 316 sites in the United States [5]. It enrolled men aged 45 to 80 years with confirmed hypogonadism (total testosterone < 300 ng/dL on two separate morning measurements) and at least one cardiovascular risk factor or established cardiovascular disease.

Participants received transdermal testosterone gel, titrated to mid-normal physiological concentrations (350–750 ng/dL), rather than supraphysiological levels. The primary endpoint was a composite of major adverse cardiovascular events (MACE): non-fatal myocardial infarction, non-fatal stroke, and cardiovascular death. The trial was event-driven, with a median follow-up of 3.2 years.

This design addressed several limitations of earlier studies: the trial was adequately powered, enrolled a population that closely reflects routine clinical practice, used adjudicated endpoints, and avoided the supraphysiological testosterone levels that had confounded the TOM trial. The choice of transdermal gel as the study formulation was deliberate, it allows more precise titration and avoids the sharp peaks associated with injectable preparations. The main characteristics of TRAVERSE and the other pivotal studies are summarised in Table 1.

**Table 1:** Pivotal Randomized Trials and Meta-Analyses on Cardiovascular Safety of Testosterone Therapy

Study (Year)	Design / Population	Testosterone Formulation	Follow-up	CV Endpoint Definition	AF / VTE / AKI Reported	Main Finding
TOM Trial (2010)	RCT; older men with mobility limitations	Transdermal gel	6 months	Composite CV events (non-adjudicated)	Not systematically assessed	Trial stopped early for excess CV events; supraphysiologic serum testosterone levels achieved
TRAVERSE (2023)	RCT; *n* = 5,246 men with confirmed hypogonadism and high CV risk	Transdermal gel (titrated to mid-normal)	Median 3.2 years	MACE: nonfatal MI, nonfatal stroke, CV death (adjudicated)	AF, PE, AKI reported	Non-inferiority vs. placebo for MACE; higher non-MACE signals with low absolute rates
Corona et al. (2014)	Meta-analysis of > 70 RCTs	Mixed formulations	≤ 3 years (most trials)	MACE / CV events (heterogeneous)	Limited reporting	No increased risk of MACE; trials underpowered for CV outcomes
Hudson et al. (2022)	IPD + aggregate meta-analysis of RCTs	Mixed formulations	Variable	MI, stroke, CV mortality	Not systematically assessed	No excess MI, stroke, or CV mortality
Corona et al. (2024)	Updated meta-analysis; 106 RCTs, > 15,000 men	Mixed formulations	Variable	MACE, MI, stroke	Limited arrhythmia data	No increased MACE irrespective of baseline CV risk
Braga et al. (2025)	Meta-analysis; 23 placebo-controlled RCTs	Mixed formulations	Longest available	Adjudicated CV outcomes	Non-sustained arrhythmias	Overall CV neutrality; modest increase in non-sustained arrhythmias

The experience of the TOM study, where supraphysiological testosterone exposure in a high-risk population led to early trial termination, directly informed the TRAVERSE protocol. By titrating to mid-normal levels and excluding men with very recent cardiovascular events, the trial was designed to test the safety of TRT as it would be used in careful clinical practice, not in an extreme scenario.

### Primary endpoint: MACE

The primary finding of TRAVERSE was straightforward: TRT was non-inferior to placebo with respect to major adverse cardiovascular events (MACE), with a hazard ratio of 0.96 (95% CI 0.78–1.17) [5]. This result was consistent across all pre-specified subgroups, including men with established cardiovascular disease, those with diabetes, and across the full age range of the study population.

Importantly, the upper limit of the confidence interval remained well below the predefined non-inferiority margin of 1.50. Taken together, these data provide reassuring evidence that, over a median follow-up of 3.2 years, TRT administered at physiological doses does not increase the risk of major cardiovascular events in this population.

The clinical implications are relevant. For many men with confirmed organic hypogonadism who have historically been denied TRT because of cardiovascular safety concerns, TRAVERSE offers a more solid basis for reconsidering treatment, provided that patient selection is appropriate and monitoring is carefully implemented. This interpretation is consistent with the individual patient data meta-analysis by Hudson and colleagues, which did not show an increased risk of cardiovascular mortality or events across 35 trials [8], as well as with the updated meta-analysis by Corona and colleagues that incorporates TRAVERSE data [9].

### The non-inferiority margin and its limitations

Before moving to the non-MACE findings, it is important to recognize what TRAVERSE cannot fully answer. Although the trial provides robust reassurance regarding major adverse cardiovascular events, the median follow-up of 3.2 years may still be too short to capture potential late cardiovascular consequences of long-term testosterone exposure. This issue is particularly relevant for outcomes such as atrial fibrillation, which may remain intermittent, asymptomatic, and clinically unrecognized for prolonged periods. In addition, only a small proportion of participants were older than 75 years of age (approximately 8%), limiting the applicability of the findings to the oldest and most vulnerable patients commonly encountered in routine clinical practice. Importantly, demonstrating non-inferiority for MACE should not be interpreted as proof of universal cardiovascular safety across all possible outcomes. While the primary endpoint was reassuring, other cardiovascular and systemic signals emerged during the study and deserve separate consideration. Contemporary guideline documents have appropriately emphasized these nuances, encouraging clinicians to interpret the reassuring MACE data within the broader context of individualized risk assessment and long-term monitoring [10, 11].

## Cardiovascular safety beyond mace

### Erythrocytosis: The most consistent signal

Among the non-MACE findings emerging from TRAVERSE, erythrocytosis represented the most consistent and clinically established adverse effect of testosterone therapy. Haematocrit levels ≥ 54% were observed in 17.0% of men receiving TRT compared with only 3.3% in the placebo group (*p* < 0.001) [5]. This effect has been repeatedly documented across randomised trials, observational studies, and virtually all testosterone formulations, and its consistency across study designs gives it a degree of clinical certainty that most other TRT-associated signals do not yet possess.

The biological mechanisms are well characterised. Testosterone acts through at least three partially independent pathways to expand red blood cell mass: it directly stimulates erythroid progenitor cells in the bone marrow via androgen receptor-mediated genomic effects; it increases renal erythropoietin production; and it suppresses hepcidin, the master regulator of iron homeostasis, thereby enhancing ferroportin-mediated iron release and increasing the availability of substrate for erythropoiesis [12]. Testosterone aromatisation to oestradiol may additionally stimulate haematopoietic stem cell proliferation, though this pathway appears to be a contributing rather than an essential mechanism [12]. The convergence of these pathways explains both the consistency and the dose-dependence of the erythrocytosis signal. The clinical magnitude of haematocrit elevation is greater than the TRAVERSE threshold figure of 17% might suggest. In a Swiss single-centre retrospective cohort of 247 patients followed for a median of 2.9 years, Neidhart and colleagues found that 57% of men reached a haematocrit above 0.46, 23% exceeded 0.50, and 5% exceeded the 0.54 threshold used in TRAVERSE, with 54% of patients reaching their peak haematocrit only after the first year of treatment, an observation with direct implications for monitoring duration [12]. The risk of erythrocytosis is not uniform across formulations. Intramuscular testosterone cypionate and enanthate produce the largest haematocrit increments (+ 4.0% points on average), while intranasal gel produces no statistically significant change; in a head-to-head randomised comparison of 39 men, polycythemia (Hct > 52%) developed in 4.2% of men receiving intramuscular testosterone cypionate and in none of those receiving nasal testosterone over four months [13, 14]. Long-acting testosterone undecanoate shows a haematocrit increment intermediate between the two extremes, consistent with its more stable pharmacokinetic profile.

Individual patient risk is not determined by formulation alone. In the Neidhart cohort, baseline haematocrit above 0.41 (OR 4.71, 95% CI 1.21–18.29) and higher BMI (OR 1.13–1.15 per unit) were independent predictors of clinically significant haematocrit elevation, while smoking showed a trend toward association that did not reach significance in that sample [12]. A clinically important and underappreciated interaction has recently been described in men with type 2 diabetes: in a retrospective cohort of 5,235 men with T2DM and hypogonadism drawn from the Clalit Healthcare Services database, concomitant use of SGLT-2 inhibitors was associated with a markedly elevated risk of haematocrit exceeding 54% during TRT (OR 4.85, 95% CI 3.06–7.69, versus OR 1.88 without SGLT-2 inhibitors; *p* = 0.02), a finding attributable to the additive erythropoietic effects of both drug classes [15]. Given the high prevalence of SGLT-2 inhibitor use in the population of men most likely to receive TRT, this interaction deserves explicit discussion during treatment planning.

The cardiovascular consequences of TRT-induced erythrocytosis remain an area of active investigation, but the available evidence is concerning. In a large matched cohort study of 5,842 men, those who developed polycythemia (Hct > 52%) during the first year of TRT had significantly higher rates of both MACE and VTE compared with matched controls who did not develop polycythemia, suggesting that erythrocytosis is not merely a laboratory finding but a mechanistically plausible intermediate on the pathway to adverse vascular events [14]. The mechanism, increased blood viscosity promoting a prothrombotic milieu, is biologically coherent and consistent with the VTE signal discussed in Sect.  4.3.

From a practical standpoint, current evidence supports haematocrit monitoring at 3–6 months during the first year of TRT and annually thereafter, with dose reduction or temporary treatment discontinuation when haematocrit exceeds 54% or haemoglobin exceeds 18.5 g/dL [12, 15]. Therapeutic phlebotomy may be considered in selected cases where treatment continuation is clinically important. Importantly, given that more than half of patients reach peak haematocrit after the first year, annual monitoring should not be interpreted as a licence to reduce vigilance. In men with borderline baseline haematocrit, OSA, COPD, or concurrent SGLT-2 inhibitor therapy, we recommend more frequent early monitoring (at one month, then three months) and a lower threshold for formulation change toward transdermal or intranasal preparations. These monitoring recommendations are reflected in Table 2.

**Table 2:** Key Clinical Messages Post-TRAVERSE

Domain	Key Clinical Messages (Post-TRAVERSE)	Supporting Evidence
Cardiovascular Safety (MACE)	TRT does not increase the risk of major adverse cardiovascular events (MACE) when prescribed to men with rigorously confirmed hypogonadism and titrated to physiological testosterone levels, including those at high baseline cardiovascular risk.	TRAVERSE, NEJM 2023; Corona et al. 2024
Non-MACE CV Events	Non-MACE safety signals (atrial fibrillation, venous thromboembolism, acute kidney injury) were numerically higher in the TRT arm in TRAVERSE, with low absolute event rates. These findings warrant vigilance rather than contraindication, particularly in men with pre-existing risk factors.	TRAVERSE, NEJM 2023; Braga et al. 2025
Hematologic Safety	Erythrocytosis represents the most consistent adverse event of TRT. Hematocrit should be monitored regularly, and TRT should be reduced or temporarily withheld if hematocrit ≥ 54%.	TRAVERSE; Endocrine Society 2018; SIAMS–SIE 2022
Prostate Safety	No excess risk of prostate cancer has been observed in RCTs and meta-analyses up to mid-term follow-up; long-term oncologic safety remains under investigation.	TRAVERSE; Corona et al. 2024
Patient Selection	TRT should be initiated only in men with biochemically confirmed hypogonadism (total testosterone < 300 ng/dL on two separate morning samples using reliable assays) and clinically relevant symptoms.	Endocrine Society 2018; SIAMS–SIE 2022
Functional Hypogonadism	Functional hypogonadism related to obesity or metabolic syndrome should prompt lifestyle and comorbidity management as first-line therapy. TRT may be considered in persistently symptomatic men after careful risk–benefit evaluation.	Grossmann 2018; Corona 2018
Baseline Evaluation	Before initiating TRT: assess hematocrit, PSA (age- and risk-appropriate), blood pressure, glycemic and lipid profile, obstructive sleep apnea risk, and personal history of venous thromboembolism or arrhythmias.	Guidelines + TRAVERSE
Follow-Up Monitoring	Reassess testosterone levels, hematocrit, symptoms, and adverse events at 3–6 months and periodically thereafter. Blood pressure and cardiovascular status should be monitored during treatment.	TRAVERSE; Guidelines
Arrhythmia Surveillance	Individualized ECG monitoring may be considered in men with prior atrial fibrillation, conduction abnormalities, or significant arrhythmic risk.	TRAVERSE; Braga et al. 2025
Regulatory Perspective	In 2025, the FDA removed the boxed cardiovascular warning previously applied to testosterone use for age-related decline, while maintaining restrictions to approved indications and emphasizing appropriate monitoring.	FDA 2025
Unresolved Issues	Key uncertainties include long-term oncologic safety, optimal duration of TRT in very old men (> 80 years), and integration of TRT into multimorbidity and frailty management pathways.	Ongoing

### Hypertension

A modest but statistically significant increase in systolic blood pressure, approximately 1.6 mmHg above placebo, was observed in the TRAVERSE trial among men receiving testosterone therapy [5]. This finding was considered clinically important enough to prompt a 2025 class-wide FDA labelling update requiring all testosterone products to include explicit warnings regarding potential blood pressure elevation (FDA, 2025, https://www.fda.gov/drugs/drug-safety-and-availability/fda-updates-labeling-testosterone-products-regarding-blood-pressure-increases). The regulatory response is proportionate: while 1.6 mmHg is a small absolute increment, it acquires clinical relevance when considered in the context of populations already carrying substantial cardiometabolic burden.

The mechanisms underlying this effect are likely multifactorial. Testosterone may promote sodium and water retention and has complex effects on endothelial function and coronary vasodilation that can be directionally opposite depending on the dose and the vascular bed involved [16]. One particularly well-characterised contributor is the erythrocytosis pathway: as haematocrit rises, blood viscosity increases, augmenting peripheral vascular resistance and amplifying the pressor response to testosterone. Olesen and colleagues demonstrated this interaction directly in a randomised trial, showing that rising haematocrit levels significantly amplified the blood pressure response to TRT in men with opioid-induced androgen deficiency [17]. This mechanistic link between erythrocytosis and hypertension reinforces the argument that several of the non-MACE signals associated with testosterone therapy are physiologically interconnected rather than independent adverse events.

The direction of the blood pressure effect is not, however, uniformly adverse. In men with metabolic syndrome, obesity, or type 2 diabetes, populations in whom hypogonadism is frequently functional rather than organic, TRT has been associated with reductions in both systolic and diastolic blood pressure in multiple interventional studies, including a 15-month trial of parenteral testosterone undecanoate and a randomised study in men with metabolic syndrome and recent-onset diabetes where testosterone produced beneficial blood pressure effects beyond those achieved by diet and exercise alone [18]. This apparent paradox likely reflects the fact that, in these patients, correcting the hypogonadal state improves the underlying metabolic milieu, reducing visceral adiposity, insulin resistance, and sympathetic tone, to an extent that outweighs any direct pressor effect of testosterone.

The epidemiological data confirm that hypertension and testosterone prescribing are closely intertwined, but the causal direction is not straightforward. In a large retrospective cohort of 20,299 men from North-West London primary care, hypertension was present in 25.7% of men who were subsequently prescribed TRT, compared with 17.8% of those who were not (OR 1.60, 95% CI 1.35–1.90, *p* < 0.0001), suggesting that hypogonadism and hypertension frequently co-occur, most plausibly because both are downstream consequences of obesity and metabolic syndrome, rather than that TRT causes hypertension in a previously normotensive man [19]. In a propensity-weighted provincial cohort of 6,949 men receiving TRT followed over a median of more than two decades in Manitoba, Canada, both TRT-treated men and untreated testosterone-deficient men showed a similarly elevated MACE risk (HR 1.27, 95% CI 1.16–1.39 and HR 1.21 respectively), a finding that underscores the contribution of the underlying hypogonadal state, and its associated cardiometabolic burden, including hypertension, to cardiovascular risk, independent of treatment [20].

From a clinical standpoint, the practical implication is one of careful patient stratification rather than categorical avoidance. In a normotensive man with organic hypogonadism and no metabolic comorbidities, the 1.6 mmHg increment observed in TRAVERSE is unlikely to alter his cardiovascular trajectory meaningfully. In a man with borderline or treated hypertension, chronic kidney disease, or established cardiovascular disease, even a small upward pressure shift may be clinically consequential over time, and blood pressure should be measured at baseline and at each monitoring visit. In men with metabolic syndrome or obesity-related functional hypogonadism, the priority should be metabolic optimisation before TRT initiation, not only because testosterone normalisation may occur spontaneously with weight loss, but because the blood pressure effects of TRT in this group may be genuinely beneficial once the metabolic substrate is adequately addressed.

### Non-MACE safety signals: a differentiated assessment

The non-MACE safety signals in TRAVERSE require careful interpretation. The trial data and the observational data tell a subtly different story, and conflating them as has sometimes been done in clinical guidelines and regulatory documents leads to either overestimation or underestimation of risk depending on which source is emphasised.

#### Atrial fibrillation (AF)

The TRAVERSE trial identified a numerical excess of atrial fibrillation in the testosterone arm, 3.1% versus 2.4% in the placebo group (HR 1.33, 95% CI 0.97–1.83, p = NS), that did not reach statistical significance but represented an absolute risk difference of 0.7% points over a median follow-up of 3.2 years [5]. The failure to reach significance is almost certainly a reflection of statistical power rather than true absence of effect: AF is a relatively rare outcome, and TRAVERSE was not designed or powered to detect small increments in arrhythmic events as a primary endpoint.

The aggregate evidence from randomised trials, while numerically reassuring, is constrained by the same limitation. A meta-analysis of 26 randomised controlled trials enrolling 10,941 hypogonadal men found a pooled relative risk of 1.38 (95% CI 0.54–3.53, *p* = 0.36) for incident AF with TRT versus placebo, a point estimate directionally consistent with TRAVERSE but with confidence intervals too wide to exclude clinically meaningful harm [16]. Critically, only 4 of the 26 included trials contributed AF data, confirming that the existing randomised evidence base is substantially underpowered for this outcome and that the absence of a statistically significant signal should not be interpreted as evidence of safety.

Observational data provide important contextual information, though they are subject to substantial confounding. A large prospective cohort study of 173,498 men followed for 11 years within the UK Biobank demonstrated a complex relationship between endogenous androgen levels and incident AF: low serum testosterone was associated with a modestly increased AF risk (HR 1.06, 95% CI 1.00–1.12), whilst elevated sex hormone-binding globulin (SHBG) conferred a more pronounced risk increment (HR 1.44, 95% CI 1.34–1.54), suggesting that the androgenic milieu modulates atrial electrophysiology through multiple pathways [21]. This finding is physiologically important: it implies that the relationship between testosterone and AF risk is not linear, and that both testosterone deficiency and the hormonal dysregulation associated with elevated SHBG may independently promote arrhythmic substrate. In a large retrospective primary care cohort of 20,299 men, those subsequently prescribed TRT had significantly higher baseline prevalence of ischaemic heart disease (OR 1.57, *p* = 0.002), obstructive sleep apnoea (OR 3.47, *p* < 0.0001), previous DVT or pulmonary embolism (OR 2.24, *p* = 0.002), and prior stroke (OR 1.82, *p* = 0.03), all established risk factor for AF, and were on average six years older with a higher BMI than untreated men [19]. This profile of pre-treatment comorbidity is a critical source of residual confounding in all observational analyses of TRT and AF, and it renders direct causal inference from observational data alone methodologically untenable.

Pharmacovigilance data from the FDA Adverse Event Reporting System (FAERS), encompassing 28,921 TRT-related reports submitted between 2004 and 2022, identified a disproportionality signal for ventricular fibrillation (reporting odds ratio 2.51, 95% CI 1.49–4.24) and cardiac arrest (ROR 1.88, 95% CI 1.51–2.35), while characterising both events as rare in absolute terms [22]. These signals are hypothesis-generating rather than confirmatory, given the well-recognised limitations of spontaneous reporting systems, including underreporting, selective reporting, and inability to establish causation, but they are consistent with the biological plausibility of testosterone-mediated effects on ventricular electrophysiology.

The mechanistic underpinning for a testosterone–arrhythmia relationship is biologically coherent. Testosterone inhibits L-type calcium channels, modulates cardiac potassium currents, and may promote atrial structural remodelling through androgenic receptor-mediated pathways [23]. Crucially, the direction of these effects may depend on the concentration and duration of exposure: the acute anti-ischaemic and vasodilatory effects of testosterone in men with coronary artery disease are well documented at physiological levels [24], whereas chronic exposure at the upper end of the normal range, which is where many men on TRT are maintained, may have different electrophysiological consequences, particularly in atrial tissue already subject to fibrotic remodelling from hypertension, obesity, or sleep apnoea.

The overall interpretation is one of a plausible but incompletely characterised signal. The trial and observational data are directionally consistent: both point toward a modest relationship between the androgenic milieu and AF risk that is likely mediated through multiple converging pathways, direct electrophysiological effects, haemodynamic consequences of erythrocytosis-driven viscosity increases, and the cardiometabolic substrate of the population most likely to receive TRT. The signal does not currently justify withholding TRT from men with well-established hypogonadism, but it does justify incorporating AF risk into the pre-treatment evaluation. Baseline cardiac rhythm assessment, at minimum a standard 12-lead ECG, should be obtained in men with established cardiovascular disease, hypertension, obesity, or obstructive sleep apnoea before initiating TRT, and the arrhythmic signal should be discussed explicitly during the informed consent process in men with pre-existing conduction abnormalities or a history of paroxysmal AF.

#### Venous thromboembolism (VTE) and pulmonary embolism (PE)

Pulmonary embolism occurred in 0.9% of men receiving testosterone and 0.5% of those receiving placebo in the TRAVERSE trial, while venous thromboembolic events overall were reported at 2.0% versus 1.5% respectively, neither difference reaching statistical significance, but both directionally consistent with a pro-thrombotic signal [5, 25]. As with atrial fibrillation, the failure to reach statistical significance reflects the trial’s primary design for MACE detection rather than the absence of a biologically meaningful effect: TRAVERSE was not powered to detect small increments in rare thromboembolic events, and these findings should be interpreted as safety signals warranting further investigation rather than as evidence of null effect.

The observational literature provides a more granular picture, though it is marked by an apparent paradox that requires careful interpretation. A systematic review and meta-analysis of 14 randomised controlled trials and 10 observational studies encompassing 314,315 men found that, in RCT-derived data, TRT was not associated with a significantly increased risk of VTE overall (OR 1.42, 95% CI 0.22–9.03, *p* = 0.71) or pulmonary embolism specifically (OR 1.38, 95% CI 0.27–7.04, *p* = 0.70) [26]. Observational data from the same meta-analysis showed a paradoxical overall reduction in VTE risk (OR 0.78, 95% CI 0.61–1.00, *p* = 0.05), almost certainly a reflection of healthy-user bias and confounding by indication in real-world prescribing, but a statistically significant increase in deep vein thrombosis specifically (OR 1.44, 95% CI 1.04–2.00, *p* = 0.03; I²=0%), a finding that persisted across sensitivity analyses [26]. The discordance between the overall VTE and DVT-specific results underscores the importance of disaggregating thromboembolic outcomes when interpreting this literature: the apparent protective signal for total VTE in observational cohorts is likely artefactual, whereas the DVT signal from studies with low heterogeneity carries greater methodological credibility.

The risk appears concentrated in the early treatment period. A large population-based case-control study by Martinez and colleagues (*n* > 19,000) reported a 63% increase in VTE risk during the first six months of therapy (HR 1.63, 95% CI 1.12–2.37), corresponding to approximately 10 additional events per 10,000 person-years, with risk normalising beyond six months [6]. Walker and colleagues confirmed an approximately twofold increase in VTE risk during the first one to three months of therapy in a case-crossover study of nearly 40,000 men, irrespective of hypogonadism status and route of administration [7]. The individual patient data meta-analysis by Hudson and colleagues across 35 trials further corroborated this early temporal clustering of thromboembolic events [8].

The mechanistic explanation for this early risk window is most plausibly erythrocytosis-driven hyperviscosity. Testosterone stimulates erythropoiesis through increased renal erythropoietin production, direct bone marrow effects, and hepcidin suppression, all of which converge to raise haematocrit within the first months of treatment. Men who develop polycythaemia (Hct ≥ 52%) during TRT experience a significantly higher risk of both MACE and VTE during the first year, and the temporal coincidence of peak erythrocytosis risk and peak VTE risk is not incidental [25]. Androgens additionally increase platelet thromboxane A₂ receptor density and formation, providing a second, haematocrit-independent pathway to a prothrombotic milieu [26]. Formulation pharmacokinetics amplify this risk further: short-acting intramuscular regimens generating supraphysiological testosterone peaks are associated with greater haematocrit increments and higher rates of cardiovascular events and hospitalisation than transdermal systems in observational cohorts, and subcutaneous testosterone enanthate autoinjectors appear to produce smaller increases in both oestradiol and haematocrit than intramuscular cypionate [25].

From a clinical management standpoint, haematocrit monitoring is the central instrument for VTE risk mitigation. Monitoring should be performed at baseline, at three to six months, and annually thereafter, with temporary treatment interruption and dose adjustment triggered by Hct exceeding 54%, or 50% according to the more conservative AACE and ICSM thresholds [25, 26]. Men with prior VTE or known thrombophilia represent a particularly high-risk phenotype in whom TRT should only be initiated after explicit shared decision-making, with an individualised monitoring plan and specialist input regarding thromboprophylaxis. The pre-treatment evaluation should also encompass obstructive sleep apnoea, chronic kidney disease, and uncontrolled hypertension, all conditions that independently amplify erythrocytosis risk and thromboembolic susceptibility. Patients should be counselled proactively on alarm symptoms, new or unexplained dyspnoea, pleuritic chest pain, or unilateral leg swelling, with a low threshold for diagnostic evaluation during the first six months of therapy. A clinically important gap in current practice is that no widely used VTE risk stratification tool including the Wells criteria, the PERC score, or the YEARS algorithm, currently incorporates testosterone replacement therapy as a contributing risk factor, an omission that warrants attention in future guideline revisions [25, 27].

#### Acute kidney injury (AKI)

Acute kidney injury was the only non-MACE endpoint to reach nominal statistical significance in TRAVERSE, occurring in 2.3% of testosterone-treated men versus 1.5% of placebo recipients, an absolute risk difference of 0.8% points that, while modest, was statistically significant (*p* = 0.048) [5]. The caveat that this finding was not adjusted for multiple comparisons is methodologically important, but the signal has since been independently replicated in a propensity-matched real-world cohort of 4,268 hypogonadal men drawn from the TriNetX global research network, in which TRT was associated with a significantly increased risk of AKI over three years of follow-up (RR 1.53, 95% CI 1.07–2.18), while no significant increase in atrial fibrillation was observed in the same dataset (RR 1.48, 95% CI 0.93–2.37) [28]. This dissociation between the AKI and AF signals in real-world data reinforces the specificity of the renal finding and argues against a generic confounding explanation.

The AKI signal is further substantiated by converging evidence from propensity-matched surgical cohorts, which provide a natural experimental context in which renal vulnerability is acutely amplified. In a national database analysis of 7,906 men undergoing total hip arthroplasty, preoperative TRT use was associated with a significantly higher rate of AKI at one year (7.6% versus 5.6%, *p* < 0.001), in addition to increased rates of deep vein thrombosis at both 90 days (2.8% versus 2.0%, *p* = 0.023) and one year (4.3% versus 3.0%, *p* = 0.002) [29]. In a parallel analysis of 13,250 men undergoing total knee arthroplasty, preoperative TRT was independently associated with AKI within 90 days (OR 1.5, 95% CI 1.2–1.8, *p* < 0.001) and pulmonary embolism (OR 1.4, 95% CI 1.0–1.8, *p* = 0.041) [30], and in a third cohort of 2,738 men undergoing total shoulder arthroplasty, TRT users had a 45% higher relative risk of AKI at one year (17.5% versus 12.1%, RR 1.45, *p* < 0.001) [31]. The consistency of the AKI signal across three independent surgical cohorts and two medical datasets spanning different patient populations, surgical stresses, and database sources, substantially strengthens the biological credibility of the association. Notably, in a smaller cohort of men undergoing rotator cuff repair, a procedure associated with lower physiological stress than major joint arthroplasty, no significant difference in 90-day AKI was observed (0.7% versus 0.5%, *p* = 0.18) [32], suggesting that the magnitude of perioperative renal stress may modulate the expression of TRT-associated renal vulnerability.

The mechanistic basis for TRT-associated AKI is multifactorial. Testosterone promotes sodium and water retention through both direct renal tubular effects and RAAS activation, increasing intraglomerular pressure and fluid loading in a manner that can precipitate AKI in men with reduced renal reserve [25]. Erythrocytosis-driven hyperviscosity impairs renal microvascular perfusion, and the peak-trough variability characteristic of short-acting intramuscular formulations may generate episodic haemodynamic stress on the renal vasculature that is attenuated by more pharmacokinetically stable transdermal or long-acting preparations [25]. Importantly, a subgroup analysis of 13,598 men undergoing radical nephrectomy, a population with inherently reduced functional renal mass, found that hypogonadism itself was associated with higher rates of postoperative AKI (25.5% versus 21.6%, *p* = 0.005), but that, among hypogonadal men, TRT use was not associated with any adverse postoperative outcome [33]. This paradoxical finding reinforces the complexity of the testosterone–kidney relationship: the baseline hypogonadal state may itself confer renal vulnerability, and TRT may not be the sole or primary driver of the observed risk in all contexts. The most clinically important challenge in interpreting the AKI signal is its apparent context-dependence. A 2025 analysis of 10,009 men with type 2 diabetes and hypogonadism reported that TRT was associated with a significantly reduced risk of AKI (HR 0.76, 95% CI 0.65–0.89, *p* < 0.001), a finding diametrically opposite to the TRAVERSE signal [34]. This apparent contradiction most plausibly reflects population heterogeneity in the renal substrate: testosterone’s haemodynamic effects on renal blood flow, including vasodilation of the afferent arteriole and attenuation of the fibrotic and inflammatory milieu of diabetic nephropathy, may be nephroprotective when the primary renal threat is ischaemic and metabolic, while the same haemodynamic and erythropoietic effects may be harmful in men whose renal vulnerability is driven by reduced nephron mass, established CKD, or perioperative stress. The implication is that the direction of the renal effect of TRT is not fixed but depends critically on the underlying renal phenotype of the patient. From a clinical management standpoint, pre-treatment renal assessment is mandatory in all men being considered for TRT, and CKD stage 3–5 (eGFR < 60 ml/min/1.73 m²), a solitary kidney, rapidly progressive CKD, or a history of recurrent AKI should each be considered high-risk phenotypes requiring intensified monitoring or reconsideration of treatment initiation [25]. Renal function should be reassessed at three to six weeks after initiation, capturing the early haemodynamic response and again at three months, with annual review thereafter in stable patients and more frequent assessment in those with baseline renal impairment. A confirmed eGFR decline of ≥ 20% from baseline should trigger evaluation for intercurrent illness, nephrotoxin exposure, and reversible contributors, with dose reduction, formulation change, or temporary interruption as appropriate; persistent or recurrent renal dysfunction should prompt formal reassessment of the indication for TRT [25]. In men with pre-existing CKD or significant proteinuria, nephrology input before initiation and at the first sign of renal deterioration is a clinically prudent step that the current evidence base supports.

#### Fractures

A 2026 meta-analysis by Anagnostis and colleagues reported an increased risk of clinical fractures in men receiving TRT (HR 1.43, 95% CI 1.04–1.97) [35], a finding that was unexpected given the well-established anabolic effects of testosterone on bone mineral density. This paradox is further supported by large observational database studies: a PearlDiver analysis of 77,491 matched pairs reported a vertebral fracture incidence of 0.31% in TRT users versus 0.04% in controls (adjusted OR 7.70, 95% CI 5.09–11.65, *p* < 0.001), corresponding to an incidence rate 7.6 times higher than in untreated men [36]; a separate PearlDiver analysis of 335,753 matched pairs reported a proximal humerus fracture incidence of 0.029% versus 0.005% (adjusted OR 3.14, 95% CI 1.84–5.59, *p* < 0.001), with risk concentrated in the 46–55 year age group (adjusted OR 7.52, 95% CI 2.47–32.73) [37]. The apparent contradiction between BMD benefit and fracture risk increase is reconciled by a possible behavioural–musculoskeletal mismatch hypothesis: TRT-related improvements in muscle strength, energy, and perceived vitality lead to increased physical activity and fall exposure in men who were previously sedentary, before slower connective tissue and bone quality adaptations have had time to fully consolidate [36, 38]. Most fractures in TRAVERSE occurred at traumatic sites (ribs, wrists, ankles) and clustered in the early months after treatment initiation, supporting a behavioural rather than a bone quality mechanism [38]. This interpretation is consistent with the high-quality RCT data from the T-Trial Bone Study, which demonstrated a lumbar spine trabecular volumetric BMD increase of 7.5% (95% CI 4.8–10.3%, *p* < 0.001) at 12 months, and the T4 Bone Trial, which showed significant cortical BMD gains at tibia and radius at 24 months, genuine skeletal benefit at the tissue level that has not yet translated into demonstrable fracture risk reduction at the clinical level in any adequately powered trial [39, 40].

It is important to note that untreated hypogonadism is itself a significant fracture risk factor (OR 1.76, 95% CI 1.37–2.26), accounting for 16–30% of male osteoporosis cases [41]. The fracture signal with TRT therefore does not argue for withholding treatment from hypogonadal men, it argues for careful activity counselling, fall risk assessment, and, where indicated, concurrent osteoporosis-specific therapy. Current Endocrine Society guidelines recommend TRT for symptomatic relief in hypogonadal men but do not endorse it as primary treatment for osteoporosis; in men with both hypogonadism and osteoporosis, TRT should be combined with evidence-based antiresorptive therapy [42]. In our practice, we have become more cautious about initiating TRT in men over 75 with a history of falls or reduced bone density, and we now routinely assess fall risk and consider DXA scanning in this group before starting therapy. Counselling on gradual activity progression avoiding an abrupt transition from sedentary to physically demanding behaviour is a practical measure that we have found valuable in reducing early injury risk.

## Organic versus functional hypogonadism

### Clinical differentiation and therapeutic implications

The distinction between organic and functional hypogonadism is not merely taxonomic; it has direct and immediate consequences for treatment decisions. In our experience managing men with hypogonadism in a tertiary endocrinology setting, failure to make this distinction is one of the most common sources of inappropriate TRT prescribing.

Organic hypogonadism reflects permanent failure of the hypothalamic–pituitary–gonadal (HPG) axis due to an identifiable structural or pathological cause, such as Klinefelter syndrome, bilateral orchidectomy, pituitary adenoma, haemochromatosis, prior chemotherapy or radiotherapy, or traumatic brain injury, among others. In these patients, testosterone will not normalise without exogenous replacement, and TRT is the appropriate and necessary treatment. Delay is not justified. Controlled interventional evidence supports the clinical efficacy of testosterone replacement in appropriately selected hypogonadal men, with benefits observed across sexual function, body composition, bone outcomes, and selected patient-reported measures [43].

Functional hypogonadism, by contrast, reflects reversible suppression of the HPG axis secondary to systemic metabolic or chronic disease, most commonly obesity, metabolic syndrome, type 2 diabetes, obstructive sleep apnoea, or chronic opioid use. In these patients, low testosterone is a consequence of the underlying condition rather than a primary endocrine failure. Treating testosterone deficiency without addressing the underlying driver is, at best, incomplete and, at worst, counterproductive.

The evidence supporting metabolic reversibility is growing. Emerging data suggest that targeted treatment of obesity may partially restore endogenous gonadal function while simultaneously improving body composition and sexual outcomes. In a recent controlled pilot study, tirzepatide treatment in men with obesity-associated metabolic hypogonadism was associated with improvements in gonadotropins, total and free testosterone, lean mass, and erectile function, supporting a pathophysiology-based rather than replacement-based therapeutic approach [44]. In a prospective cohort of 64 men with male obesity secondary hypogonadism (MOSH) who underwent bariatric surgery, testosterone levels normalised in 81.4% at 6 months and 89.5% at 24 months [45]. Liraglutide 3.0 mg daily normalised testosterone and improved sexual function in obese men with functional hypogonadism who had been poor responders to lifestyle measures, with a mean weight loss of 7.9 ± 3.8 kg compared with 0.9 ± 4.5 kg in the TRT arm (*p* < 0.001) [46].

The data on combining TRT with lifestyle intervention are equally instructive, and perhaps more cautionary. A 2025 randomised trial by Gonzalez-Gil and colleagues compared intensive lifestyle intervention alone versus intensive lifestyle intervention plus TRT in 120 men with functional hypogonadism and metabolic syndrome. At 12 months, the addition of TRT did not improve glycaemic control (HbA1c change − 0.5% versus − 0.6%, *p* = 0.35) and, unexpectedly, blunted the rise in HDL cholesterol that would normally accompany successful lifestyle intervention (HDL change 0.2 ± 1.1 versus 5.4 ± 1.0 mg/dL, *p* = 0.01) [47]. This finding suggests that adding TRT prematurely may actually interfere with the metabolic benefits of lifestyle change, rather than augmenting them.

A recent head-to-head comparison provides further evidence for prioritising metabolic intervention in men with obesity-related functional hypogonadism who desire fertility. In a 24-week randomised trial of 25 men with type 2 diabetes, obesity, and functional hypogonadism, semaglutide 1 mg weekly significantly improved sperm morphology (from 2% to 4% morphologically normal sperm, *p* = 0.012) and increased total testosterone, whereas intramuscular TRT improved erectile function but significantly decreased sperm concentration [48]. Both groups experienced improvements in symptoms of hypogonadism as measured by the Aging Symptoms in Men (AMS) score, but semaglutide was clearly superior for men prioritising fertility preservation.

The role of TRT in functional hypogonadism is not, however, limited to cases where metabolic intervention has failed. A growing body of evidence supports TRT as a complementary strategy to lifestyle modification, rather than a substitute for it. A 2024 meta-analysis by Corona and colleagues demonstrated that TRT combined with lifestyle intervention increases muscle mass and facilitates physical exercise, thereby improving compliance with lifestyle programs [49]. Among dietary interventions, very low-calorie ketogenic diets (VLCKD) showed the best results for testosterone normalisation; among exercise modalities, aerobic or endurance training was particularly effective in older men. The authors concluded that TRT should be viewed as a valuable complementary strategy to increase muscle mass and facilitate physical exercise while improving sexual symptoms, thus favouring motivation and compliance for lifestyle interventions.

This complementary role is particularly relevant in real-world practice, where sustained weight loss is difficult to achieve. As Shenoy and colleagues noted in a 2024 review of MOSH management, weight loss is the ideal therapeutic strategy, but achievement of significant weight reduction is usually difficult with lifestyle changes alone in real-world practice [50]. TRT is often necessary when lifestyle measures fail, and it improves many comorbidities related to obesity. The authors emphasised that balancing the risks and benefits of TRT should be considered in every patient before and during long-term management.

The evidence for metabolic benefits of TRT in functional hypogonadism is substantial. In a 9-month randomised trial of 80 men with functional hypogonadism and type 2 diabetes, transdermal TRT significantly reduced waist circumference, HOMA-IR, and HbA1c (*p* < 0.001, *p* = 0.002, and *p* = 0.004, respectively), as well as markers of endothelial dysfunction including leptin, resistin, CRP, ICAM-1, and p-selectin [51]. Advanced age attenuated these effects, whereas higher delta-testosterone levels amplified them, suggesting that achieving adequate testosterone increments is critical for metabolic benefit. Corona and colleagues argued in 2018 that the significant improvements in body composition, glucose metabolism, and sexual activity justify the use of TRT not only in organic hypogonadism but also in functional hypogonadism [52].

Current European Academy of Andrology (EAA) guidelines reflect this evolving understanding. Lifestyle changes and weight reduction should be the first approach in all overweight and obese men with functional hypogonadism [53]. Transdermal testosterone should be preferred for initiation of TRT. Gonadotrophin therapy is recommended only when fertility is desired in men with secondary hypogonadism. TRT is able to reliably improve global sexual function in hypogonadal men in the short term, though longer-term clinical benefits and safety remain to be fully documented.

### Alternatives to TRT in functional hypogonadism

For men with functional hypogonadism in whom TRT is contraindicated or undesirable, several alternative pharmacological approaches have been explored, though the evidence base remains limited.

Gonadotropins (hCG and FSH) are effective in restoring endogenous testosterone production and preserving or restoring fertility in men with central hypogonadism, but they require frequent injections and are more expensive than TRT [54]. They are the treatment of choice when fertility is desired in the near future.

Selective estrogen receptor modulators (SERMs) clomiphene citrate and tamoxifen have been used off-label for functional hypogonadism. Several studies report significant increases in testosterone levels with these agents, but their use is off-label and data supporting efficacy on hypogonadal symptoms are insufficient [54]. A 2020 systematic review concluded that SERMs might be an alternative for TRT, but more research is needed to evaluate their effect on hypogonadal signs and symptoms, as well as their long-term safety profile [55]. Aromatase inhibitors (AIs) are not recommended in functional hypogonadism because of insufficient efficacy and a decrease in bone mineral density.

GLP-1 receptor agonists represent a particularly promising alternative in obese men with functional hypogonadism. As discussed above, semaglutide and liraglutide not only normalise testosterone but also improve metabolic parameters, reduce weight, and in the case of semaglutide improve sperm morphology [46, 48]. For men with obesity-related functional hypogonadism who desire fertility, GLP-1 agonists should be considered first-line therapy.

### Functional hypogonadism in specific clinical contexts

Several clinical contexts require tailored approaches to functional hypogonadism.

Depression: Depressive disorders are a recognised cause of functional hypogonadism, and the relationship is bidirectional. Men with hypogonadism can manifest depressed mood, anxiety, insomnia, and memory impairment. Mild depressive symptoms may respond to TRT, but severe depressive symptoms do not respond to TRT [56]. The prevalence of functional hypogonadism in depression, and of depressive symptoms in hypogonadal men, is not well established, and the clinical relevance of each condition to the other remains to be clarified. In practice, we assess mood systematically in all men presenting with hypogonadism, and we involve psychiatry colleagues when depressive symptoms are moderate or severe.

Older men: The diagnosis of functional hypogonadism in older men should prompt a thorough assessment and optimisation of general health, including lifestyle changes, weight reduction, care of comorbidities, and cessation of offending medications, some of which can lead to meaningful gains in endogenous testosterone concentrations [57]. Having excluded or addressed reversible causes and contraindications, patients with functional hypogonadism can be offered a trial of TRT after full discussion on the anticipated benefits and potential risks. TRT improves libido but may be less effective for erectile dysfunction. TRT can also have modest positive effects on insulin resistance, bone strength, some measures of physical strength, and mild depressive symptoms, but the clinical significance of these relatively short-term improvements remains uncertain in terms of longer-term patient-important outcomes. Initiation of TRT is a joint decision between patient and clinician, since longer-term benefits and risks have not been adequately defined.

Heart failure: Functional hypogonadism is common in heart failure (HF), affecting up to 90% of patients as part of multiple hormonal deficiency syndrome (MHDS), and is associated with worse outcomes. A 2025 systematic review of randomised controlled trials in HF patients reported that TRT improved muscle strength and aerobic capacity, increased lean muscle mass, decreased fat mass, improved insulin sensitivity, and shortened the QT interval [58]. TRT did not consistently affect blood pressure, lipid profiles, or heart rate, nor did it lead to any serious adverse effects. However, the current evidence is limited by small sample sizes and short follow-up periods. Larger event-driven RCTs evaluating hard endpoints are needed to determine whether TRT should be integrated into standard HF therapies.

Fertility preservation: For men with functional hypogonadism who desire fertility, TRT is contraindicated because it suppresses spermatogenesis. Gonadotropins are the treatment of choice in this setting [54]. GLP-1 agonists, particularly semaglutide, represent a valuable alternative that can improve both testosterone levels and sperm quality while addressing the underlying metabolic dysfunction [48].

### Clinical recommendation

In men with functional hypogonadism, metabolic optimisation should precede TRT. A trial of lifestyle intervention targeting ≥ 5–10% weight loss with or without GLP-1/GIP agonist therapy, should be attempted for at least 3–6 months before TRT is considered. If testosterone normalises and symptoms resolve, TRT is not required. If testosterone remains low and symptoms persist after adequate metabolic intervention, TRT may be considered as a complementary strategy to facilitate exercise, improve muscle mass, and enhance compliance with ongoing lifestyle modification. The indication for TRT should be reassessed at each subsequent follow-up visit, particularly if metabolic parameters are improving.

Table 3 provides a structured comparison of organic and functional hypogonadism across ten clinical parameters.

**Table 3:** Organic vs. Functional Hypogonadism: Clinical Characteristics and Therapeutic Implications

Parameter	Organic Hypogonadism	Functional Hypogonadism
**Definition**	Permanent failure of the hypothalamic-pituitary-gonadal (HPG) axis due to an identifiable structural or pathological cause	Reversible suppression of the HPG axis secondary to systemic metabolic or chronic disease; no primary gonadal or pituitary pathology
**Common Causes**	• Klinefelter syndrome • Bilateral orchidectomy / cryptorchidism • Pituitary adenoma or hypopituitarism • Haemochromatosis • Chemotherapy / radiotherapy • Traumatic brain injury	• Obesity / metabolic syndrome • Type 2 diabetes mellitus • Obstructive sleep apnoea • Chronic inflammatory disease • Opioid use / glucocorticoid therapy • Severe psychological stress
**Gonadotropins** **(LH / FSH)**	Primary: elevated (hypergonadotropic) Secondary: low or normal (hypogonadotropic)	Low or normal (hypogonadotropic) — reflects central suppression, not gonadal failure
**Testosterone Level**	Persistently low; unlikely to normalise without TRT	Low, but potentially reversible with metabolic optimisation
**Reversibility**	Generally irreversible; lifelong TRT usually required	Potentially reversible with lifestyle intervention, weight loss, and comorbidity management
**First-Line Management**	TRT (after exclusion of contraindications and after shared decision-making with the patient)	Lifestyle modification: weight loss, increased physical activity, dietary optimisation, management of contributing comorbidities • GLP-1/GIP agonists (e.g., tirzepatide: T ↑ from 322 ± 111 to 380 ± 130 ng/dL, *p* < 0.001; Alexopoulos, 2024, doi:https://doi.org/10.1111/dom.15612) • Bariatric surgery (MOSH remission: 81.4% at 6 months, 89.5% at 24 months; Pellitero, 2012, doi:https://doi.org/10.1007/s11695-012-0734-9) • Optimisation of OSA, glycaemic control, chronic disease
**Role of TRT**	Indicated as primary treatment in confirmed symptomatic patients without contraindications	Consider TRT only in persistently symptomatic men after ≥ 3–6 months of adequate metabolic optimisation, following careful risk-benefit evaluation and a joint clinical decision
**Caution: TRT + Lifestyle**	—	Adding TRT to intensive lifestyle intervention does not improve glycaemic outcomes (HbA1c − 0.5% vs. − 0.6%, *p* = 0.35) and may blunt metabolic benefits (HDL rise 0.2 ± 1.1 vs. 5.4 ± 1.0 mg/dL, *p* = 0.01) (Gonzalez-Gil, 2025, doi:https://doi.org/10.1210/clinem/dgae249)
**Monitoring Priorities**	Haematocrit, PSA, testosterone levels, symptoms, blood pressure; ECG if arrhythmic risk	Testosterone levels, metabolic parameters (HbA1c, lipids, BMI, waist circumference), symptoms; reassess indication for TRT at each follow-up
**Key Clinical Message**	TRT is the appropriate and necessary treatment; delay is not justified in confirmed organic hypogonadism	TRT is not the first step; metabolic optimisation should precede pharmacological therapy and may render TRT unnecessary

BMI = body mass index; FSH = follicle-stimulating hormone; GLP-1 = glucagon-like peptide-1; GIP = glucose-dependent insulinotropic polypeptide; HbA1c = glycated haemoglobin; HDL = high-density lipoprotein; HPG = hypothalamic-pituitary-gonadal; LH = luteinising hormone; MOSH = male obesity secondary hypogonadism; OSA = obstructive sleep apnoea; PSA = prostate-specific antigen; TRT = testosterone replacement therapy

## Discussion and conclusions

Translating the aggregate trial evidence into decisions for individual patients requires a framework that goes beyond population-level risk estimates. The men who present to endocrinology clinics with hypogonadism are rarely the uncomplicated, relatively healthy men who populated the TRAVERSE trial they have prior atrial fibrillation, prior VTE, chronic kidney disease, uncontrolled hypertension, or a history of prostate cancer in remission. For these patients, the TRAVERSE MACE data provide limited direct guidance, and clinical decision-making must integrate trial evidence, observational signals, mechanistic plausibility, and individual risk phenotypes.

Three points deserve particular emphasis when managing high-risk scenarios. First, the absolute contraindications are genuinely absolute: active prostate cancer, male breast cancer, recent MI or stroke within three to six months, severe decompensated heart failure, baseline haematocrit exceeding 50%, and desire for fertility preservation [59]. These are not situations where a careful risk-benefit discussion might justify TRT — they are situations where TRT should not be initiated. Second, prior atrial fibrillation and prior VTE are not absolute contraindications, but they require specialist input before TRT is started [60, 6, 7]. A cardiologist should be involved in the AF decision; a haematologist or anticoagulation specialist in the VTE decision. The key question in both cases is whether the underlying arrhythmia or thrombotic risk is adequately managed, and whether the erythrocytosis risk of TRT can be monitored and controlled. In our own practice, we have found that joint clinics with cardiology colleagues lead to better outcomes and greater patient confidence than sequential consultations. Third, the older and frailer the patient, the more individualised the decision must be. Only 8% of TRAVERSE participants were over 75 years of age, and the safety of TRT in men over 80 remains essentially unknown [5]. The fracture signal (HR 1.43) is particularly relevant in this group [35]. Functional goals like muscle strength, quality of life, sexual function must be weighed against safety uncertainties that current evidence cannot fully resolve. Table 4 provides a structured framework for risk stratification across these and other clinical scenarios. Structured monitoring is not optional; it is the mechanism by which the non-MACE signals identified in TRAVERSE can be detected and managed before they become clinically significant. At three to six months, total testosterone, haematocrit, PSA, blood pressure, and symptoms should be assessed; annually thereafter, these parameters plus lipid profile, HbA1c, and a clinical review of the TRT indication. If haematocrit reaches or exceeds 54%, dose reduction or temporary withholding is required, with investigation of contributing factors such as obstructive sleep apnoea, COPD, or dehydration [61, 62]. If PSA rises more than 1.4 ng/mL within 12 months, urology referral is indicated [10]. ECG monitoring should be considered in men with prior AF or significant arrhythmic risk [60]. In functional hypogonadism, the TRT indication should be reassessed at every follow-up visit [63]. On the regulatory front, 2025 brought a significant shift. The FDA removed the boxed cardiovascular warning previously applied to testosterone products prescribed for age-related decline, whilst maintaining restrictions to confirmed hypogonadism and requiring structured monitoring (FDA, 2025). The EMA position has similarly evolved toward careful patient selection rather than blanket restriction (EMA, 2024). These regulatory changes reflect the cumulative weight of evidence, with TRAVERSE as its centrepiece, and provide a clearer framework for prescribers who had previously been operating under warnings that were not commensurate with the available data. Several limitations of this review and of the underlying evidence base deserve explicit acknowledgement. This is a narrative review, not a systematic review or meta-analysis, and the synthesis inevitably involves interpretive judgements that a formal meta-analytic approach would handle differently. TRAVERSE itself has important limitations: median follow-up of 3.2 years may be insufficient to detect late cardiovascular effects; only 8% of participants were over 75; and the exclusive use of transdermal testosterone gel means MACE non-inferiority cannot be extrapolated to injectable formulations [5, 64]. Observational data on non-MACE signals are subject to residual confounding, as men prescribed TRT may differ systematically from those not prescribed it in ways that are difficult to fully adjust for [6, 7]. Evidence for TRT in functional hypogonadism remains limited, with most trials short-term and conducted in selected populations [43, 45]. These limitations do not invalidate the clinical recommendations offered in this review, but they define the boundaries within which those recommendations should be applied.

**Table 4:** High-Risk Clinical Scenarios: Recommendations for TRT Use

Clinical Scenario	Caution Level	Evidence Base	Recommendation	Monitoring / Action
Absolute Contraindications				
**Active or suspected prostate cancer**	**Contraindicated**	Testosterone is a known growth promoter for androgen-sensitive prostate cancer; excluded from all major RCTs	TRT is absolutely contraindicated. Urological evaluation required before any future consideration	PSA + urological assessment before any TRT initiation
**Male breast cancer (active)**	**Contraindicated**	Androgen-sensitive malignancy; no RCT data available	TRT is absolutely contraindicated	Oncology consultation required
**Recent MI or stroke** **(< 3–6 months)**	**Contraindicated**	Excluded from TRAVERSE; no safety data in acute post-event period (Endocrine Society 2018)	TRT should not be initiated within 3–6 months of acute MI or stroke. Reassess after cardiovascular stabilisation	Cardiology consultation before any TRT consideration
**Severe heart failure** **(NYHA class III–IV)**	**Contraindicated**	Excluded from TRAVERSE; limited safety data; fluid retention risk (Endocrine Society 2018)	TRT is contraindicated in decompensated or severe heart failure	Cardiology review; optimise HF therapy first
**Haematocrit > 50%** **at baseline**	**Contraindicated**	Excluded from TRAVERSE; TRT-induced erythrocytosis would further increase thrombotic risk	Do not initiate TRT until haematocrit is < 50%. Investigate and treat underlying cause	Repeat haematocrit; investigate secondary erythrocytosis (OSA, COPD, myeloproliferative disease)
**Desire for fertility** **preservation**	**Contraindicated**	Exogenous testosterone suppresses spermatogenesis via HPG axis suppression	TRT is contraindicated in men wishing to preserve fertility. Consider clomiphene citrate or hCG-based therapy	Semen analysis; andrology/reproductive medicine referral

High Caution — Relative Contraindications				
**Prior VTE / PE** **or thrombophilia** **[Corrected]**	**High Caution**	Observational data: Martinez 2016 (OR 1.63, 95% CI 1.12–2.37; population-based case-control, *n* > 19,000) Walker 2020 (large US cohort, *n* > 39,000) Hudson 2022 (IPD meta-analysis) Risk highest in first 6 months; mechanism: erythrocytosis-induced hyperviscosity	Shared decision-making essential. Consider anticoagulation status, thrombophilia screening, and alternative treatments. If TRT initiated, start at lowest effective dose	Haematocrit at 1, 3, 6 months; thrombophilia screen if not done; educate patient on VTE symptoms
**Prior atrial fibrillation** **[Corrected]**	**High Caution**	TRAVERSE: AF 3.1% vs. 2.4% (HR 1.33, 95% CI 0.97–1.83, p = NS) Observational data — Xu 2024 (UK Biobank, *n* = 173,498, 11 years): HR 1.06 for low T (95% CI 1.00–1.12) HR 1.44 for high SHBG (95% CI 1.34–1.54) Braga 2025 meta-analysis: modest signal for non-sustained arrhythmic events	Individualised risk-benefit assessment with cardiologist. If TRT initiated, ensure rate/rhythm control is optimised	ECG at baseline and 3–6 months; symptom review at each visit; cardiology co-management recommended
**Uncontrolled hypertension** **(BP > 160/100 mmHg)**	**High Caution**	Excluded from TRAVERSE; TRT associated with mean SBP increase of + 1.6 mmHg; FDA class-wide labelling change (2025) (Olesen, 2024, doi:https://doi.org/10.1097/HJH.0000000000003638)	Optimise blood pressure control before initiating TRT. If BP > 160/100 mmHg, defer TRT until controlled	BP at every visit; intensify antihypertensive therapy if sustained elevation; monitor haematocrit (BP and Ht are linked)
**Untreated or severe** **obstructive sleep apnoea**	**High Caution**	Testosterone may worsen sleep-disordered breathing; OSA is also a risk factor for erythrocytosis during TRT (Hoyos, 2012, doi:https://doi.org/10.1111/j.1365-2265.2012.04413.x)	Initiate CPAP therapy and optimise weight management before or concurrent with TRT. Re-evaluate OSA severity after 3 months of TRT	Repeat sleep study if symptoms worsen; haematocrit monitoring (OSA increases erythrocytosis risk); weight monitoring

Moderate Caution — Use After Careful Risk–Benefit Assessment				
**Chronic kidney disease** **(CKD stage ≥ 3)**	**Moderate Caution**	TRAVERSE: AKI 2.3% vs. 1.5% (*p* = 0.048; not adjusted for multiple comparisons) Bonnet 2025: reduced AKI risk in diabetic men (HR 0.76, 95% CI 0.65–0.89) Effect may be population-specific	Assess baseline eGFR and proteinuria. If CKD stage ≥ 3, initiate TRT at low dose with nephrology input	eGFR and creatinine at 3 months, then every 6 months; BP monitoring; avoid concurrent nephrotoxic drugs
**Established CV disease** **(stable; no recent events)**	**Moderate Caution**	TRAVERSE specifically enrolled this population: MACE HR 0.96 (95% CI 0.78–1.17) — reassuring for MACE; non-MACE signals persist (Lincoff, 2023, doi:https://doi.org/10.1056/NEJMoa2215025)	TRT can be considered after shared decision-making addressing both MACE safety and non-MACE signals (AF, VTE, AKI). Cardiology input recommended	ECG at baseline; haematocrit, BP, renal function at 3–6 months; annual cardiovascular review
**Older men (> 75 years)** **/ frail patients**	**Moderate Caution**	Only 8% of TRAVERSE participants were > 75 years; fracture HR 1.43 (95% CI 1.04–1.97; Anagnostis 2026); safety in men > 80 years unknown	Individualised risk-benefit assessment mandatory. Discuss uncertainty regarding long-term outcomes. Consider functional goals (muscle strength, QoL) vs. safety concerns	Fall risk assessment; bone density (DEXA) if osteoporosis risk; haematocrit; cognitive and functional status at each visit
**Treated low-risk prostate** **cancer (in remission)**	**Moderate Caution**	No excess prostate cancer in TRAVERSE or meta-analyses; however, long-term oncologic safety unconfirmed; limited RCT data in this subpopulation	Multidisciplinary consultation (urology/oncology) required. TRT may be considered after ≥ 1–2 years of disease-free follow-up in selected patients	PSA every 3–6 months for first 2 years; DRE annually; urology co-management mandatory
**Type 2 diabetes /** **metabolic syndrome** **(functional hypogonadism)**	**Moderate Caution**	TRT added to lifestyle intervention did not improve HbA1c (− 0.5% vs. − 0.6%, *p* = 0.35) and blunted HDL rise (0.2 ± 1.1 vs. 5.4 ± 1.0 mg/dL, *p* = 0.01) (Gonzalez-Gil, 2025, doi:https://doi.org/10.1210/clinem/dgae249)	Prioritise metabolic optimisation (lifestyle, GLP-1/GIP agonists, bariatric surgery) for ≥ 3–6 months before considering TRT. Reassess testosterone after weight loss	HbA1c, lipids, BMI, waist circumference at each visit; reassess testosterone level and TRT indication after metabolic intervention

Use with Structured Monitoring — Standard Risk				
**Confirmed organic** **hypogonadism**,** no major** **comorbidities**	**Use with Monitoring**	TRAVERSE: MACE HR 0.96 (95% CI 0.78–1.17); non-inferiority confirmed in men with confirmed hypogonadism and elevated CV risk (Lincoff, 2023, doi:https://doi.org/10.1056/NEJMoa2215025)	TRT is appropriate after biochemical confirmation (testosterone < 300 ng/dL ×2), symptom assessment, baseline evaluation, and shared decision-making	Testosterone, haematocrit, PSA at 3–6 months; BP at each visit; annual review thereafter
**Erythrocytosis during** **TRT (Ht 50–54%)**	**Use with Monitoring**	Erythrocytosis occurred in 17.0% vs. 3.3% in TRAVERSE; risk factors include older age, OSA, COPD, injectable formulations (Fink, 2025, doi:https://doi.org/10.1093/sxmrev/qeaf013; Bachman, 2014, doi:https://doi.org/10.1093/gerona/glt154)	Reduce TRT dose or switch to transdermal formulation. Consider therapeutic phlebotomy if Ht persists > 54%. Withhold TRT if Ht ≥ 54% until resolved	Haematocrit monthly until stable; investigate contributing factors (OSA, COPD, dehydration); consider switch to lower-dose transdermal formulation

Scenarios are classified by caution level based on available evidence from TRAVERSE, observational studies, and current guideline recommendations

AF = atrial fibrillation; AKI = acute kidney injury; BP = blood pressure; CI = confidence interval; CKD = chronic kidney disease; COPD = chronic obstructive pulmonary disease; CPAP = continuous positive airway pressure; CV = cardiovascular; DEXA = dual-energy X-ray absorptiometry; eGFR = estimated glomerular filtration rate; HbA1c = glycated haemoglobin; HF = heart failure; HR = hazard ratio; Ht = haematocrit; MACE = major adverse cardiovascular events; MI = myocardial infarction; NYHA = New York Heart Association; OSA = obstructive sleep apnoea; PE = pulmonary embolism; PSA = prostate-specific antigen; QoL = quality of life; SBP = systolic blood pressure; TRT = testosterone replacement therapy; VTE = venous thromboembolism.

TRAVERSE has substantially clarified the cardiovascular risk landscape of TRT in older men with confirmed hypogonadism. The demonstration of non-inferiority for MACE outcomes (HR 0.96, 95% CI 0.78–1.17) [5], across a broad range of baseline cardiovascular risk, provides a sound evidence base for treatment decisions that had previously rested on extrapolation and regulatory caution. The non-MACE signals (atrial fibrillation, venous thromboembolism, acute kidney injury, erythrocytosis) require a more nuanced interpretation. They are real, directionally consistent across trial and observational data, and carry mechanistic plausibility. But there are not reasons to withhold TRT from patients who genuinely need it, they are reasons to select patients carefully, monitor systematically, and adjust treatment when monitoring reveals a problem.

The organic versus functional distinction is the single most important clinical decision in male hypogonadism management. Prescribing TRT to a man whose testosterone is low because of obesity and metabolic syndrome, without first attempting metabolic intervention, is not evidence-based practice. The data from tirzepatide trials [43], bariatric surgery cohorts [45], and the Gonzalez-Gil trial [47] make this point with increasing clarity. Metabolic optimisation should precede TRT in functional hypogonadism, and the indication should be reassessed at each follow-up visit as metabolic parameters improve.

The questions that remain unanswered - long-term oncological safety, TRT in men over 80, confirmation of the fracture signal, outcomes with non-transdermal formulations - are real and important. They will require prospective trials with longer follow-up, broader age ranges, and more diverse formulations than TRAVERSE provided. Until those trials are completed, we must work within the boundaries of the evidence we have, applying it with clinical judgement, humility, and a willingness to revisit our decisions as new data emerge.
